# Factors affecting the economic burden of breast cancer in southern Iran

**DOI:** 10.1186/s12913-023-10346-5

**Published:** 2023-12-01

**Authors:** Faride Sadat Jalali, Mozhgan Seif, Abdosaleh Jafari, Vahid Zangouri, Khosro Keshavarz, Ramin Ravangard

**Affiliations:** 1 Student Research Committee, School of Health Management and Information Sciences, Shiraz University of Medical Sciences, Shiraz, Iran; 2 Non-communicable Disease Research Center, Department of Epidemiology, School of Health, Shiraz University of Medical Sciences, Shiraz, Iran; 3 Health Human Resources Research Centre, School of Health Management and Information Sciences, Shiraz University of Medical Sciences, Shiraz, Iran; 4 Breast Diseases Research Center, Shiraz University of Medical Sciences, Shiraz, Iran

**Keywords:** Breast neoplasms, Cost of Illness, Economic burden, Cost driver, Iran

## Abstract

**Background:**

Breast cancer (BC) is the most common cancer in the world, and is associated with significant economic costs for patients and communities. Therefore, the information on the costs of the disease and the identification of its underlying factors will provide insights into designing effective interventions and reducing the costs. Thus, the present study aimed to identify the factors affecting the economic burden of breast cancer from all medical centers providing diagnostic and treatment services in southern Iran.

**Methods:**

A list of factors affecting the economic burden of breast cancer was obtained based on the effective factors searched in the databases, including PubMed, ProQuest, Scopus, ISI Web of Science, SID, and Magiran, and the opinions of BC cancer specialists. Then, the data on 460 breast cancer patients was collected from March 2020 to March 2022. The relationship between the factors affecting Breast Cancer costs was analyzed using SPSS 13.0 software by the use of multiple regression analysis.

**Results:**

The results of the multiple regression analysis showed that stages (P-value < 0.001), being an extreme user (p = 0.025), type of treatment center (P-value < 0.001), income (P-value < 0.001), chemotherapy side effects (P-value < 0.001), and distance to the nearest health center (P-value < 0.001) were important factors affecting the costs of breast cancer patients.

**Conclusions:**

According to the results, encouraging people to undergo annual screenings, increasing insurance coverage, assuring the patients about the desirability and adequacy of the provided medical services, deploying specialists in chemotherapy centers (especially nutritionists) to recommend special diets, and establishing cancer diagnostic and treatment centers in high-population cities could help reduce the costs of breast cancer patients.

**Supplementary Information:**

The online version contains supplementary material available at 10.1186/s12913-023-10346-5.

## Introduction

Breast cancer (BC) is the most common cancer worldwide [1], and is more frequently diagnosed in women [2] than in men [3]. The disease occurs when breast cells begin to grow uncontrollably. BC can be diagnosed through mammography, specialized X-ray scans, or by finding an abnormal mass in the breast tissue, either by the patient or their doctor [4]. Many risk factors contribute to the development of BC, including early menstruation, late menopause, marriage at an older age, use of contraceptives, breastfeeding, inactivity and obesity, family history, history of radiation exposure, and alcohol and tobacco use. The risk of infection increases with increasing age [2, 5, 6]. According to the latest reports from the World Health Organization, in 2020, 2.3 million women were diagnosed with BC, and approximately 30% of them died [7]. In the United States, BC was diagnosed in 287,850 women and 2,710 men in the first six months of 2022, and 4,3780 of them died [2, 8, 9]. Additionally, the latest statistics published by the Institute for Health Metrics and Evaluation (IHME) and the Global Burden of Disease (GBD) studies indicate that in 2019, there were 498,000 new cases of breast cancer in Europe, resulting in approximately 157,000 deaths, 19,268 cases in Australia with 4,481 deaths, 181,500 cases in Africa with 58,700 deaths, and 864,000 cases in Asia with 257,000 deaths [10].

Along with the increase in the number of cancer cases, the costs associated with cancer have also significantly increased worldwide. In most developed countries, cancer results in a significant increase in national economic burden [11–13] and is associated with significant economic costs for patients, payers, and communities. At the end of 2021, the annual cost of healthcare systems related to breast cancer was approximately 20 billion dollars worldwide [13]. A study conducted by the Health Promotion Center at the University of North Carolina (UNC) suggests that BC costs will significantly increase in the next decade, depending on trends in incidence, progression, survival, and prevention of the disease. Researchers estimate that by 2030, the costs of BC worldwide will reach 152.5 billion dollars [14]. The treatment burden for BC patients receiving care includes direct payments for health services, such as high out-of-pocket expenditures (i.e., co-payments and high deductibles), and indirect costs secondary to lost productivity and wages [13].

Considering the fact that the data used to develop medical guidelines in high-income countries [15] are often inapplicable to patients in low- and middle-income countries (LMIC) due to economic and biological differences, the WHO and the prevailing health policies in such countries have called for an increase in cancer treatment and research efforts in low- and middle-income countries along with the increased incidence of cancer in these regions [16]. Since 2013, low- and middle-income countries have taken important steps in developing capacity for BC diagnosis and treatment, including the development of cancer centers in resource-limited regions and providing wide access to low-cost drugs and new diagnostic technologies [17]. In addition, since the beginning of the last century, countries around the world have started research on the economic burden of cancer. The main purpose of the economic burden studies is to assess the economic burden that diseases impose on individuals or communities [18]. Economic burden studies convert disease-related burdens into economic and monetary values to measure socioeconomic costs unavoidably incurred by a given community in terms of certain diseases [19]. The economic burden of disease includes direct and indirect costs that affect the individual’s quality of life, and the imposed costs cause significant psychological pain for the patient and their family [20]. Therefore, having information about the costs of diseases and the influential factors is necessary for the formulation and prioritization of healthcare policies and the efficient allocation of scarce resources [21]. Furthermore, identifying the underlying drivers of high economic burden provides insights into designing effective and appropriate interventions to meet the needs of costly diseases and reduce their costs [22]. Some studies, including those conducted in Iran, have shown that factors affecting the costs of BC include the type of treatment and the stage of the disease, use of chemotherapy drugs, presence of comorbidities, increasing age, education level, receiving radiotherapy treatment, obesity, smoking, undergoing surgery, number of hospitalization days, and health insurance status and type of basic and supplementary insurances [22–29].

Effectively and reasonably controlling the growth of medical costs is of great significance in reducing the economic burden of disease on society. However, the management of breast cancer in Iran is currently too extensive, which is not conducive to the reasonable control of expenses. Therefore, it has become an urgent and realistic research topic to explore the important factors that affect the costs of breast cancer patients and provide a scientific basis for establishing a reasonable reimbursement mechanism and standard for the expenses of breast cancer patients. The researchers did not find any study that comprehensively addressed the factors affecting the direct and indirect costs of breast cancer, especially in Iran. Therefore, the present research aimed to investigate the factors affecting the economic burden of breast cancer in Iran. It is hoped that the results of this research will help healthcare providers identify these factors and plan interventions to reduce the economic burden of breast cancer in the future.

## Methods

To identify the factors influencing the costs of BC, the researchers first conducted a scoping review of previous studies and consulted with BC specialists to identify the most influential factors. Then, they determined the relationship between these factors and the costs of the disease.

### Identifying the factors affecting the costs of BC through the literature review

This part of the study was conducted in six stages based on the scoping review method of Joanna Briggs Institute (JBI) as a framework. In the first stage, a question was raised based on the Population (or participants)/Concept/Context (PCC) elements was raised. All the patients with BC (population), the factors affecting BC costs (concept), and all countries of the world (context) were included in the question. The target population included all studies on the “factors affecting BC costs” in various countries. This part of the study was a scoping review, based on the method of Joanna Briggs Institute (JBI) as a framework. Scoping reviews can determine the main components and related aspects of a specific concept, and thus, help to draw a thematic map based on the collected evidence and identify the knowledge gaps in the scope. Thus, determining the factors affecting economic burden of BC was performed in the following six stages: In the first stage, a specific question was asked based on the PCC elements (population, content, and context) as follows: All the patients with BC (population), the factors affecting BC economic burden (concept), and all countries of the world (context). The research question was “What are the factors affecting BC patients economic burden in various countries?

In the second stage, all relevant studies conducted until 2021 were retrieved through the search strategy (Table 1). In the third stage, the research team selected the main keywords related to the research objective. The fourth stage involved searching for relevant articles in the intended databases including PubMed, ProQuest, Scopus, ISI Web of Science, SID, and Magiran. For this study, all articles with at least an English abstract listed in one of the intended databases until 25 December 2021 were searched and retrieved (Table 1). Inclusion criteria were articles with at least one English abstract indexed in one of the mentioned databases, which pointed to factors affecting the economic burden of BC based on selected keywords and their synonyms. Exclusion criteria included letters to editors, commentaries, and types of reviews and those studies that had not been published by the time of the study. The indexed information of the studies in the mentioned databases was transferred to the EndNote software with the help of keywords and the relevant studies were selected according to the purpose of the research. Qualitative evaluation, in addition to the selection of related articles and extraction of their data, was performed by two researchers separately. Selected articles were qualitatively evaluated by researchers using the STROBE (Strengthening the Reporting of Observational Studies in Epidemiology) checklist (https://www.strobe-statement.org/).

**Table 1:** The search strategy of the research

Database	Search string	Number of retrieved papers	Limits
**Scopus**	TITLE-ABS (neoplasm OR cancer) AND TITLE-ABS (breast) AND TITLE-ABS (cost OR expenditure OR “cost of illness” OR “cost analysis” OR economics OR “burden of illness” OR “economic burden” OR “illness burden” OR “direct cost” OR “indirect cost” OR “financial burden”) AND TITLE-ABS (factor OR variable OR determinant OR agent OR driver) AND (LIMIT-TO (OA, “all”)) AND (LIMIT-TO (DOCTYPE, “ar”)) AND (LIMIT-TO (LANGUAGE, “English”))	1343	Language (only resources with at least an abstract in English), searching the keywords in the title and abstract. Date: up to 25 December 2021
**PubMed**	(((neoplasm[Title/Abstract] OR cancer[Title/Abstract]) AND (breast[Title/Abstract])) AND (cost[Title/Abstract] OR expenditure[Title/Abstract] OR “cost of illness“[Title/Abstract] OR “cost analysis“[Title/Abstract] OR economics[Title/Abstract] OR “burden of illness“[Title/Abstract] OR “economic burden“[Title/Abstract] OR “illness burden“[Title/Abstract] OR “direct cost“[Title/Abstract] OR “indirect cost“[Title/Abstract] OR “financial burden“[Title/Abstract])) AND (factor[Title/Abstract] OR variable[Title/Abstract] OR determinant[Title/Abstract] OR agent[Title/Abstract] OR driver[Title/Abstract])	1422	
**WOS**	AB= ((neoplasm OR cancer) AND (breast) AND (cost OR expenditure OR “cost of illness” OR “cost analysis” OR economics OR “burden of illness” OR “economic burden” OR “illness burden” OR “direct cost” OR “indirect cost” OR “financial burden”) AND (factor OR variable OR determinant OR agent OR driver))) *AND* **LANGUAGE**: (English) *AND* **DOCUMENT TYPES**: (Article) Indexes = SCI-EXPANDED, SSCI, A&HCI, CPCI-S, CPCI-SSH, BKCI-S, BKCI-SSH, ESCI Timespan = All years	3343	
	TI= ((neoplasm OR cancer) AND (breast) AND (cost OR expenditure OR “cost of illness” OR “cost analysis” OR economics OR “burden of illness” OR “economic burden” OR “illness burden” OR “direct cost” OR “indirect cost” OR “financial burden”) AND (factor OR variable OR determinant OR agent OR driver))) *AND* **LANGUAGE**: (English) *AND* **DOCUMENT TYPES**: (Article) Indexes = SCI-EXPANDED, SSCI, A&HCI, CPCI-S, CPCI-SSH, BKCI-S, BKCI-SSH, ESCI Timespan = All years	90	
	Combine TI And Ab Search results with OR	3433	
**ProQuest**	Ab (neoplasm OR cancer) AND ab (breast) AND ab (cost OR expenditure OR “cost of illness” OR “cost analysis” OR economics OR “burden of illness” OR “economic burden” OR “illness burden” OR “direct cost” OR “indirect cost” OR “financial burden”) AND ab (factor OR variable OR determinant OR agent OR driver)	433	
	ti(neoplasm OR cancer) AND ti(breast) AND ti(cost OR expenditure OR “cost of illness” OR “cost analysis” OR economics OR “burden of illness” OR “economic burden” OR “illness burden” OR “direct cost” OR “indirect cost” OR “financial burden”) AND ti(factor OR variable OR determinant OR agent OR driver	4	
**SID**	Cancer (N: 2371)	After Screen: 47	
	Cancer (in Persian) (N: 6929)		
**Magiran**	Breast Cancer (N: 3049)	After Screen: 52	
	Breast cancer (in Persian) (N:2650)	After Screen: 24	

It is worth mentioning that all research processes and article selection were performed independently by two researchers, and a third researcher was responsible for reaching the consensus if necessary. Finally, the data were entered into the data extraction form based on the intended information (see Table S1 in the Appendix).

### Completing the list of searched influential factors on BC costs using the opinions of BC specialists

In the next step, the researchers provided the list of factors affecting BC costs found in the articles to BC specialists and asked them to suggest any additional factors. This process continued until saturation of the factors was reached. The characteristics of the eleven specialists participating in the study are presented in Table 2.

**Table 2:** Characteristics of breast cancer specialists

Row	Type of specialization	N
1	Blood and Cancer Specialist (Hematology-Oncology)	3
2	Surgical Oncology Fellowships	4
3	Assistant Professor of Social Medicine - Member of Breast Diseases Research Center	1
4	Radiation oncologist	2
5	Radiologist and breast cancer specialist	1

### Determining the relationship between the influential factors found in the articles and suggested by the specialists and the costs of BC

#### Sample size

In order to calculate the required sample size to estimate the mean of healthcare cost among Iranian patients with breast cancer, the result of the study by Davari et al. [30] was applied. According to this study the standard deviation of the cost was S = 0.63. Therefore by substituting in the following formula, 230 BC patients were required as the samples for each of the years 2021 and 2022, assuming $\alpha =0.05, \text{and}\, d=0.06$.

$$n=\frac{{z}_{1-\frac{\alpha }{2}}^{2}\times {s}^{2}}{{d}^{2}}$$

Where: n = sample size, z = level of confidence according to the standard normal distribution (for a level of confidence of 95%, z = 1.96), d = tolerated margin of error, and s = standard deviation.

In order to select the samples, breast cancer patients from all medical centers providing diagnostic and treatment services in southern Iran were selected using the list of patients provided by the Cancer Registry Center. These centers included hospitals, clinics, laboratories and offices in the public and private sectors. It should be noted that prevalence-based and bottom-up approaches were respectively used to prepare the cost data and calculate the costs from the societal perspective. The data on direct medical costs (DMC) were collected using the information in the patients’ medical and financial records. On the other hand, the data on direct non-medical (DNMC) and indirect costs (IC) were obtained using self-reports by the patients or their companions. Table S2 in the Appendix shows the average DMC, DNMC, and IC per studied breast cancer patient.

The collected data were then entered into the SPSS 13.0 software. The Independent sample T-test and ANOVA were used to investigate the difference in the factors affecting the costs of BC, and the Mann-Whitney test was also used for the variables of undergoing surgery and tumor recurrence, which did not have a normal distribution. The Kolmogorov-Smirnov test was used to check normality. In order to find out the correlation between some continuous variables and the cost of the healthcare, Pearson or Spearman correlations were applied; in the way that if normality was established, Pearson correlation was used and Spearman correlation was used otherwise. The correlation coefficients were also tested to assess if there was a significant correlation between covariates. Furthermore, simple and multiple analyzes were used to investigate the relationship between the factors affecting the costs of the disease. The simultaneous effect of independent variables on the disease costs was determined using the multiple linear regression model through the stepwise method. At each step, the independent variable not yet included in the regression equation with the smallest p-value was added if its p-value was smaller than 0.05. Variables already in the regression equation were removed if their p-values were larger than 0.10. It should be noted that tolerance was also checked for all predictors and if the tolerance of a variable was more than 0.0001, the variable would be a candidate for entering the model through the stepwise method. Based on the stepwise approach, the final set of predictors was selected, and a full model including all two-way interaction effects was fitted. The significance of the interaction effects was then examined. Finally, for other analyses except for multiple regression, the significance level was considered lower than 0.05.

### Costs

The cost of each patient was calculated by adding up his/her DMC, DNMC, and IC. The data collection form included four sections as follows:

#### 1) Demographic characteristics:

Demographic information of the patients were collected by reviewing the patients’ medical records.

#### 2) Direct medical costs:

The DMC of each patient were collected using a researcher-made checklist and referring to the medical centers under study. which were collected using the patients’ medical and financial records.

The total annual DMC of each patient = (average number of visits per year × visit tariff) + (average number of diagnostic-therapeutic services per year × tariff for each service) + (average number of hospitalizations per year × tariff for each day of hospitalization) + (the number of medications and drugs prescribed in a treatment period × the cost of each medication and drug unit).

#### 3) Direct non-medical costs:

To estimate the DNMC during the study period, in addition to the reports from patients or their companions, the approved government tariffs for the costs of accommodation, food, gas, and transportation expenses were used.

Average cost per patient = number of visits to receive medical services per year × cost of each visit.

#### 4) Indirect costs:

The data on the IC were collected through telephone interviews with the patients or their companions. The IC included the costs of productivity loss due to the disease (morbidity costs) and due to premature death (mortality costs). To calculate the IC, the human capital method was used, which estimates the economic value of lost productivity due to illness or death. The individuals’ wages were used to calculate lost income.

The references used to value tariffs, wages, etc. were extracted from the Ministry of Cooperatives Labor and Social Welfare and tariffs for diagnostic and treatment services in the public and private sectors [31, 32]. The costs were determined in the US dollar (USD), which, according to the website of the Central Bank of Iran (CBI), was equal to 42,000 Rials in the study years (2021 and 2022) in Iran [33].

### Ethical considerations

This study was approved by the Ethics Committee of Shiraz University of Medical Sciences (Code: IR.SUMS.REC.1400.052). The patients were free to choose to participate in the study, and once the objectives of the research were explained to them, their written informed consent was obtained. The questionnaires and checklists were completed anonymously and the patients were assured of the confidentiality of their answers to the questions. It should be noted that in order to comply with ethical considerations and the confidentiality of patient information, the patients were distinguished by the codes at the top of the data collection form.

## Results

### Identified influencing factors on BC costs through literature review

**Fig. 1:**
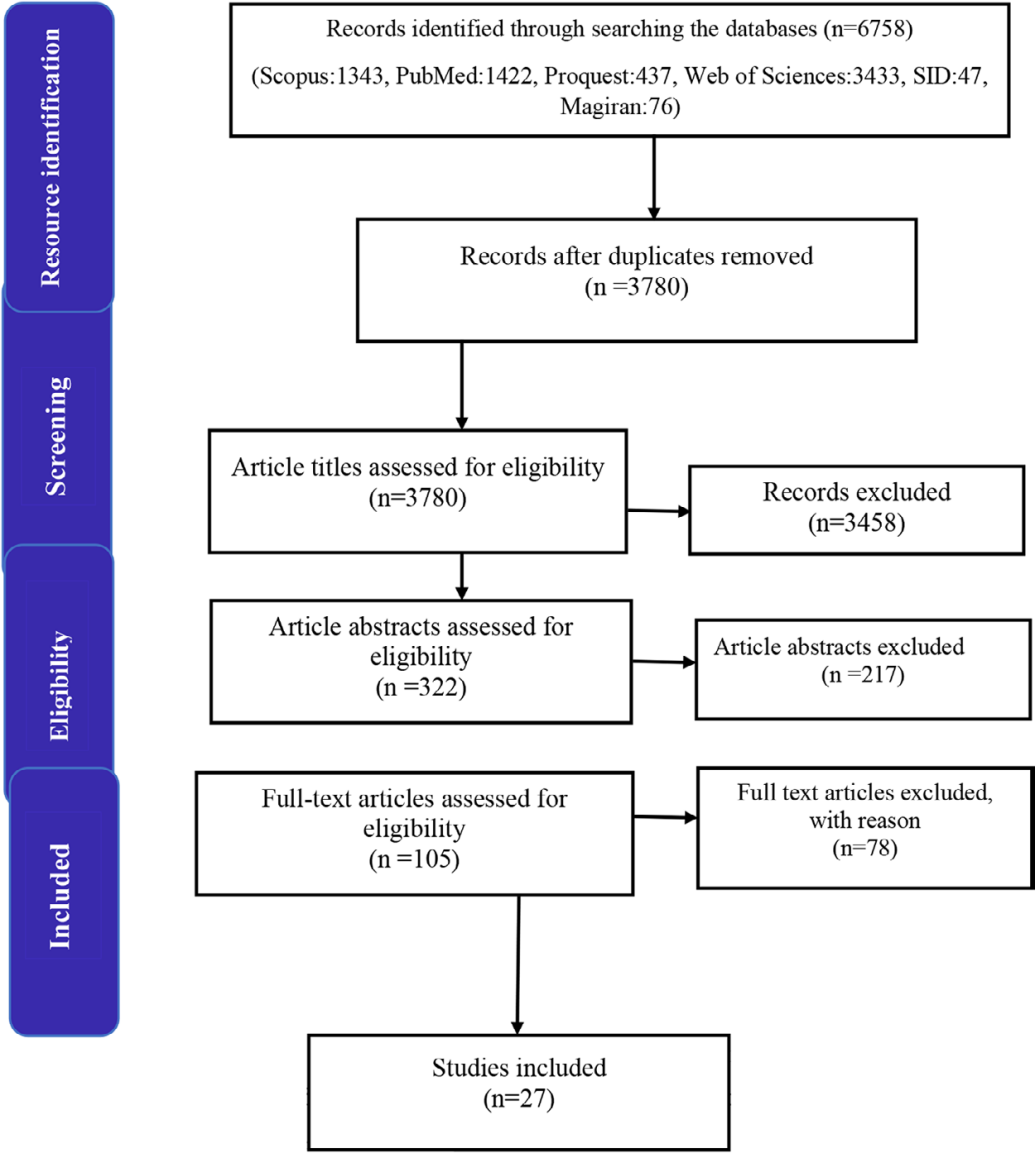
The PRISMA flow diagram for the scoping review process

The results of the search for articles in databases showed that there were 6,758 articles in the field of study purpose, of which 1,834 were duplicates. After reviewing the titles and abstracts of the remaining 3,780 English-language articles, 3,458 and 217 articles were removed from the list, respectively. A total of 105 articles were selected for full-text review. Finally, the research team selected 27 articles that answered the research question (see Fig. 1). Table S1 in the Appendix summarizes the characteristics of all 27 selected articles, including their year of publication, place (country), type of article, participants, and main results of the studies.

In the initial search, 6758 articles were identified. After removing duplicates, 3,780 articles were selected. After screening articles based on titles and abstracts, 105 articles were selected for full-text review. Finally, the research team selected 27 articles that addressed the research question. Table 3 presents the factors affecting BC costs, obtained from the literature review and suggestions from specialists. The results were obtained by examining the patients in terms of the influential factors identified.

**Table 3:** Effective factors of the costs of the breast cancer patients

Effective factors from the review of previous studies	Factors recommended by specialists
Age	
Academic Education	
Radiotherapy	Patient disability due to cancer
Stage	Diagnosis Imaging Services
Medications and drugs	Physiotherapy
Physical Activity	Hormone therapy
Supplementary Insurance	Consulting
Income level	Mastectomy
Undergoing surgery	Lymphedema
Length of stay	Chemotherapy side effects
Chemotherapy	Tumor recurrence
Smoking	Laboratory tests
Marital status	Sending samples abroad
Obesity	Babysitter care costs
Access to care	Absence from work due to the disease
Commuting for treatment	Palliative care
Social and community support	Accommodation
Type of treatment center	Patients and their companions’ food
Employment status	Early death
Diagnosis time	Waiting time
Underlying condition	
Being an extreme user	
Hospitalization	
Physician and specialist visit cost	

### Demographic characteristics of the studied Breast cancer patients

The mean age of the participating patients was 49.4 ± 10.09 years, and most of them were female (99.57%), married (78%), without academic education (67%), overweight or obese (73%), diagnosed with the disease on time (95%), underwent surgery (94%), and experienced chemotherapy side effects (59%). Additionally, most of the patients (73%) visited both private and public centers simultaneously to receive diagnostic and treatment services. Although all the patients had basic insurance coverage (100%), only 49% of them were covered by supplementary insurance organizations. A majority of the patients had no comorbidities (60%), no history of smoking (91%), no history of tumor recurrence (94%), low physical activity (46%), stage III of the disease (51%), and an income level of $1,428.57-$1,904.76 (48%). It is worth noting that the average distance from each patient’s residence to the medical centers receiving services was estimated at 100.36 ± 112 km (refer to Tables 4 and 5).

**Table 4:** Demographic Characteristics of the studied breast cancer patients (n = 460)

Characteristics		Number of patients (%)	Characteristics		Number of patients (%)
Sex	Male	2 (0.43)	Type of medical center	Public	124 (29.96)
	Female	458 (99.57)		Public- Private	336 (70.04)
Marital status	Married	359 (78.04)	Having supplementary health insurance coverage	Yes	227 (49.35)
	Single	101 (21.96)		No	233 (50.65)
Age groups (years)	28–42	124 (26.96)	BMI	< 18.5	5 (1.08)
	42–64	298 (64.78)		18.5–24.9	116 (25.22)
	64≤	38 (8.26)		25-29.9	231 (50.22)
Education level	Illiterate	18 (3.92)		30<	108 (23.48)
	Lower than diploma	294 (63.91)	Stages	I	32 (6.96)
	Academic degrees	148 (32.17)		II	97 (21.09)
Smoking	Yes	42 (9.13)		III	234 (50.86)
	No	418 (90.87)		IV	97 (21.09)

**Table 5:** The relationship between predictor factors and costs of breast cancer using linear regression analysis

Discrete Variable											
Factors		Frequency	%	Costs Mean ± SD	P-value	Simple regression			Multiple regression		
						Coefficient B	Std. Error	P-value	Coefficient B	Std. Error	P-value
Marital status	Married	357	78%	13042± 9587	0.32*	10321	1037	0.32			
	Single (ref)	103	22%	12010 ± 8084							
Academic Education	Yes	150	33%	14041 ± 11670	0.08*	1826	919	**0.05**			
	No	310	67%	12215 ± 7808							
Body Mass Index	Underweight and healthy range	122	27%	12521 ± 7843	0.71*	255	108	0.88			
	Overweight and obese range	338	73%	12851 ± 9685							
	No	305	66%	12844 ± 9781							
Diagnosis time	On-time (ref)	436	95%	12865 ± 9433	0.86***	-1044	1946	0.59			
	Late	24	5%	11822 ± 5664							
Tobacco	Yes	42	9%	14857 ± 9252	0.13*	2252	1499	0.13			
	No	418	91%	12605 ± 9261							
Physical activity	Low	212	46%	12813 ± 7927	0.98**	49.60	558	0.93			
	Moderate	153	33%	12714 ± 11506							
	High	95	21%	12961 ± 8047							
Stages	I	31	7%	8022 ± 6974	< 0.001**	3090	503	**< 0.001**	2081	465	**< 0.001**
	II	98	21%	10337 ± 7222							
	III	233	51%	12675 ± 8230							
	IV	98	21%	17146 ± 12042							
Comorbidity	Yes	182	40%	11865 ± 7534	0.06*	-1565	882	0.08			
	No	278	60%	13430 ± 10219							
Being an extreme user	Yes	19	4%	19251 ± 19377	0.02***	6717	2152	**0.002**	4312	1912	**0.02**
	No	441	96%	12533 ± 8513							
Type of treatment center	Public	123	27%	8613 ± 5271	< 0.001*	5730	941	**< 0.001**	3720	875	**< 0.001**
	Public- Private	337	73%	14343 ± 9929							
Covered by supplementary insurance	Yes	227	49%	13669 ± 10730	0.05*	1694	862	**0.05**			
	No	233	51%	11975 ± 7521							
Undergoing surgery	Yes	432	94%	13129 ± 9273	< 0.001***	5220	1794	**0.004**			
	No	28	6%	7909 ± 7916							
Tumor recurrence	Yes	28	6%	11948 ± 9242	0.26***	-919	1810	0.61			
	No	432	94%	12867 ± 9283							
Income level	I(< 952.38$)	51	11%	9112 ± 4866	< 0.001**	3780	483	**< 0.001**	2936	461	**< 0.001**
	I (952.38-1428.57$)	129	28%	10463 ± 6435							
	II (1428.57-1904.76$)	222	48%	12539 ± 8301							
	III (> 1904.76$)	58	13%	22327 ± 13943							
Chemotherapy side effects	Yes	271	59%	14575 ± 9211	< 0.001*	4293	857	**< 0.001**	3032	774	**< 0.001**
	No	189	41%	10282 ± 8785							
**Continuous Variable**											
**Factors**	**Mean ± SD**	**Spearman correlation coefficient**			**p-value**	**Simple regression**			**Multiple regression**		
						**Coefficient B**	**Sta. Error**	**P-value**	**Coefficient B**	**Sta. Error**	**P-value**
Distance to the Nearest Health Centre	100.36 ± 112	0.24			**< 0.001**	10.11	3.84	**< 0.001**	13.32	3.37	**< 0.001**
Age	49.4 ± 10.09	-0.06****			0.19	-56	0.89	0.19			

*T-Test, **ANOVA, ***Mann-Whitney U Test, **** Pearson correlation coefficient (USD $)

### Significance of relationships between identified effective factors on the economic burden of BC

The study’s results showed a significant relationship between the stage of the disease (P-value < 0.001), being an extreme user of services (P-value = 0.020), type of medical center (P-value < 0.001), having supplementary insurance coverage (P-value = 0.051), undergoing surgery (P-value < 0.001), household income level (P-value < 0.001), and chemotherapy side effects (P-value < 0.001) and the costs of BC patients. Additionally, the Spearman correlation test results in Table 5 confirmed a significant relationship between the average distance from the patient’s residence to the centers providing medical services and the costs imposed on the patient and their family (P-value < 0.001).

### Simple linear regression analysis results

The results of the simple regression analysis in Table 5 show that the costs of the disease increased with the stage of the disease, in the way that for each level increase in disease stage the cost of treatment would increase $3090 (P-value < 0.001). Being an extreme user also increased the average cost of the patients by $6,717 (P-value = 0.002). Visiting both private and public health centers simultaneously increased the cost of the patients by an average of $5,730 (P-value < 0.001). Patients who were members of supplementary insurance organizations had $1,694 more cost than other patients (P-value = 0.05). Undergoing surgery and chemotherapy side effects also increased the costs by $5,220 (P-value = 0.004) and $4,293 (P-value < 0.001), respectively. It is worth noting that in this study, the higher the household income, the greater the patients’ costs on average (an average cost of $9,112 and $22,327 in the first and fourth income levels, respectively) (P-value < 0.001).

### Multiple linear regression analysis results

The results of the simple regression analysis in Table 5 show that patients with academic education had $1,826 more costs than other patients (P-value = 0.048). Additionally, the costs of the disease increased with the stage of the disease, with fourth-stage patients spending an average of $9,124 more on their disease (P-value < 0.001). Being an extreme user also increased the average cost of the patients by $6,718 (P-value = 0.002). Visiting both private and public health centers simultaneously increased the cost of the patients by an average of $5,730 (P-value < 0.001). Patients who were members of supplementary insurance organizations had $1,694 more cost than other patients (P-value = 0.050). Undergoing surgery and chemotherapy side effects also increased the costs by $5,220 (P-value = 0.004) and $4,293 (P-value < 0.001), respectively. It is worth noting that in this study, the higher the household income, the greater the patients’ costs on average (an average cost of $9,112 and $22,327 in the first and fourth income levels, respectively) (P-value < 0.001).

The results of the multiple regression analysis showed a significant relationship between disease stage and disease costs. An increase in the stage of the disease significantly increased the costs of BC by $2,081 (P-value < 0.001). Being an extreme user, referring to both private and public centers, increased income level, and chemotherapy side effects also increased the costs of the patients by $4,312 (P-value = 0.025), $3,720, $2,936, and $3,032, respectively (all P-values < 0.001). As observed in Table 5, for every kilometer increase in the distance between the patient’s residence and medical centers, the patients’ costs increased by $10.11.

It is worth mentioning that the overall significance of the multiple regression model was also assessed and found to be significant (P-value < 0.001). Moreover, the normality of the residuals, which indicated whether they followed a normal distribution, was also confirmed (P-value = 0.30).

Overall, the results of the Independent sample T-test, ANOVA, Mann-Whitney U test, and simple and multiple regression analyses were almost consistent in terms of significance of affecting variables including: disease stage, being an extreme user, visiting both private and public centers, increased income level, and chemotherapy side effects.

The results of Table S2 (Appendix file) show that the total cost of the disease is equal to 11787.86 dollars, of which the share of DMC, DNMC and IC has been 70.48%, 19%, and 10.53%, respectively. Also, in the DMC, the largest share of costs was related to Radiotherapy (37.14%).

## Discussion

The incidence and prevalence of BC have increased in recent decades due to the progressive nature of this disease and the aging of the population [34]. The impact of this disease is evident not only in terms of mortality and complications but also in economic outcomes for all health systems and communities [8]. The aim of this study was to determine the factors affecting the costs of BC.

Based on the simple regression analysis, the results showed that having academic degrees, an increase in disease stage, being an extreme user, receiving care from both private and public service providers, having supplementary insurance coverage, undergoing surgery, experiencing chemotherapy side effects, and having higher household income led to an increase in the costs of BC patients.

The results of the multiple regression analysis in this study revealed a significant relationship between disease stage and disease costs so that higher stages of the disease significantly increased the costs. This suggests that the overall average cost was higher in higher stages of BC due to the use of more resources, longer hospitalization, more visits to cancer clinics, and the need for more care at home. In fact, the disease stage at diagnosis was an important predictor of treatment costs. Treatment for the more advanced and higher-stage disease was often more complex or invasive than treatment for earlier stages. However, higher stages not only required the use of more financial and non-financial resources but also usually had poorer health outcomes [35]. Hence, it can be stated that patients with advanced disease received more complex treatments such as chemotherapy and radiotherapy than those in the early stages. In addition, drug therapy was typically the most expensive part of treatment for patients in higher stages due to the prescription of more expensive drugs. The results of the studies by Alefan et al. (2020) in Jordan [36], Blumen et al. (2016) in the United States [37], Mittmann et al. in Canada (2014) [38], and Davari et al. in Iran (2013) [30] demonstrated that the costs of BC increased with the increase in the stage of the disease, which confirm the findings of the present study.

Being an extreme user was another effective factor found to affect the costs of BC patients in this study. Self-referred patients who visited diagnostic and treatment centers outside of the times set by physicians and specialists within a year had higher payments and, consequently, greater costs compared to other patients. The reason for being an extreme user of health services could be attributed to factors such as older age, having supplementary insurance coverage, higher education level, higher income, urbanization and shorter distance to diagnostic and treatment centers [39–41]. Accordino et al. (2017) conducted studies in the United States (2017) [42] and Colombia (2016) [43], and reported that the total cost of care for patients who were considered extreme users was higher than other patients, which are in line with the finding of the present study.

The results of the present study also showed that referring to both private and public centers led to an increase in the costs of the patients. Patients who went to private centers to receive diagnostic or treatment services had higher costs than those who received all necessary services only in public centers. This may be due to the higher service tariffs in private centers and the lack of insurance coverage for some services in these centers. Additionally, having supplementary insurance coverage may increase the probability of selecting private service providers, further contributing to the higher costs.

Slavova et al. (2020) conducted a study on cancer patients in Australia [44] and Afkar et al. (2021) [45] conducted a study in Iran comparing the costs of BC patients in public and private hospitals and concluded that the average total direct cost of the patients referred to private hospitals was higher than that of the patients who referred to public ones. This is in line with the results of the present study. However, in the study by Afkar et al. (2021) in Iran, the average total indirect cost of patients referred to private hospitals was lower than that of those referred to public hospitals. This may be due to the shorter hospitalization time of the patients in private centers, resulting in lower absenteeism from work for both patients and their companions [45].

According to the findings of this study, household income level could be another important factor contributing to the increased costs of illness. As the income level of the patient’s family increased, the costs of the disease also increased. This may be due to the fact that as household income and purchasing power increased, patients were more likely to seek care at private medical centers and use more expensive transportation and accommodation, leading to higher costs. The results of this study are similar to those of other studies, including those by Adanu et al. (2022) in Ghana [46], Sun et al. (2021) in China [47], Jing et al. (2020) in China [48], and Pisu et al. (2017) in the United States [49]. Bucknor et al. (2017) conducted a study in New York and found that patients with higher incomes used more services, often due to insurance coverage that covered most of the costs, as well as their ability to afford services [50].

The results of this study showed that chemotherapy side effects may increase the costs of patients. Patients in this study reported suffering from complications such as anemia, itchy and dry skin and nails, hair loss, dizziness, physical weakness, and sometimes nausea during their chemotherapy process. Therefore, they had to consult specialists such as dermatologists and hematologists, take medication to alleviate the side effects, or be absent from work for a short period of time, all of which incurred costs. Consequently, the costs of patients who experienced chemotherapy side effects were significantly higher than those who did not. These findings are consistent with those of other studies, including Rashid et al. (2016) [51], Hurvitz et al. (2014) [52], and Hansen et al. (2014) [53].

The results of the multiple regression analysis revealed that the cost of the disease increased as the distance from the patient’s residence to the medical center increased. This may be due to travel costs, accommodation costs, and the increased need for patient and companions’ absenteeism. Other studies, including Bona et al. (2021) [54], Slavova et al. (2020) [44], Twahir et al. (2019) [55], and Ambroggi et al. (2015) [56] also stated that increasing the distance from a patient’s residence to diagnostic and treatment centers can cause a delay in diagnosis and treatment of the disease, which in turn can lead to increased costs for BC patients [53].

The present research has some limitations, including the reliance on self-reports of patients or their companions regarding direct non-medical and indirect costs which may be subject to recall bias and imprecision. Additionally, incomplete information in some patients’ medical records and non-cooperation from some patients in providing detailed information on costs may have affected the accuracy of the data. Also, we only examined the factors that were based on the review of previous studies and derived from the opinions of breast cancer experts. Therefore, there may be other effective factors that were not included in our research. Despite these limitations, the present study provides valuable insights into the various factors that contribute to the costs of BC patients, which can help inform efforts required to improve the affordability and accessibility of cancer care.

## Conclusions

The results of this study identified several factors that were found to be influential in affecting the costs of BC. These factors included higher disease stages, being an extreme user, seeking care at both private and public health centers, experiencing chemotherapy side effects, higher levels of household income, and greater distance to medical centers. Based on the results of this study, promoting and training individuals to undergo monthly self-examinations and annual screenings before menopause could aid in the early detection of masses in lower stages, ultimately leading to reduced costs associated with BC. Furthermore, increasing basic and supplementary insurance coverage and educating patients about the quality and availability of services provided in diagnostic and treatment centers could help reduce the frequency of patients’ visits to these centers, thereby controlling costs. Moreover, the deployment of specialists, particularly nutritionists, in chemotherapy centers to provide dietary recommendations could help prevent chemotherapy side effects and reduce subsequent costs. In addition, the construction of cancer diagnosis and treatment centers in higher-population cities throughout provinces could facilitate access to services and prevent patients from having to travel long distances, thereby reducing costs associated with travel and accommodation.

### Electronic supplementary material

Below is the link to the electronic supplementary material.

Supplementary Material 1: Detailed information on selected studies & Average direct medical, direct non-medical, and indirect costs per studied breast cancer patient (USD)

## Data Availability

The data used and analyzed in the study are available from the corresponding author on reasonable request.

## Abbreviations

BC: Breast cancer
WHO: World Health Organization
IHME: Institute for Health Metrics and Evaluation
GBD: Global Burden of Disease
UNC: University of North Carolina
HIC: High-Income Countries
LMIC: Low- and Middle-Income Countries
JBI: Joanna Briggs Institute
PCC: Population (or participants)/Concept/Context
CBI: Central Bank of Iran
USD: US dollar
