# Supplementary material for: Factors affecting the economic burden of breast cancer in southern Iran
Source: BMC Health Serv Res. 2023 Dec 1;23:1332. doi: 10.1186/s12913-023-10346-5 (PMC10691120; doi:10.1186/s12913-023-10346-5)
Supplement: Supplementary file 1 — Supplementary Material 1: Detailed information on selected studies & Average direct medical, direct non-medical, and indirect costs per studied breast cancer patient (USD) [file 12913_2023_10346_MOESM1_ESM.docx]

**Appendix:**

**Table S1. Detailed information on selected studies**

| **Factors affecting the economic burden of breast cancer patients** | **type of costs** | **Participants** | **Type** | **Place** | **Year** | **Author** | **Title** | **N** |
| --- | --- | --- | --- | --- | --- | --- | --- | --- |
| age, education level, receiving radiotherapy treatment | Disease costs included periodical visits, diagnostic services, hospitalization care, treatment and rehabilitation services, home, and informal care | 138 women with breast cancer | Original Article | Iran | 2021 | Ahmadi et al | Incidence of household catastrophic and impoverishing health expenditures among patients with Breast Cancer in Iran (1) | 1 |
| ‌advanced stage.  medication costs (86.0% of total spending) | Direct medical costs (Medication, Diagnostic tests, Radiation, Hospitalization, Procedures) | 300 patients in different breast cancer stages | Original Article | Saudi Arabia | 2021 | Alghamdi et al | The Economic Burden Associated with the Management of Different Stages of Breast Cancer: A Retrospective Cost of Illness Analysis in Saudi Arabia (2) | 2 |
| lack of physical activity | Direct medical costs (Hospitalization, Chemotherapy, Radiotherapy) | 61900 patients | Original Article | Brazil | 2021 | Rezende et al | Economic burden of colorectal and breast cancers attributable to lack of physical activity in Brazil (3) | 3 |
| education, disease course, health insurance, treatment method, and income were significant predictors of CHE. | Direct medical costs  (Surgery, Radiotherapy,  chemotherapy) | 639 participating households with breast cancer patients | Original Article | China | 2021 | [Sun](https://www.ncbi.nlm.nih.gov/pubmed/?term=Sun%20Cy%5BAuthor%5D&cauthor=true&cauthor_uid=34291034) et al | Catastrophic Health Expenditure and Its Determinants Among Households with Breast Cancer Patients in China: A Multicentre, Cross-Sectional Survey (4) | 4 |
| the number of surgeries and actual hospitalization days was the important influencing factors. | Direct medical costs (Diagnostic fee, Nursing expenses,Surgical expenses, Nonsurgical expenses, Medical expenses, Cost of blood products, Cost of medical materials) | breast cancer patients in hospital | Original Article | China | 2021 | Zhang | Analysis of Influencing Factors on Hospitalization Expenses of Patients with Breast Malignant Tumor Undergoing Surgery: Based on the Neural Network and Support Vector Machine (5) | 5 |
| the invasive nature of the tumor, a mastectomy, a surgery revision, chemotherapy | Indirect costs (lost working days costs) | 604 patients with breast cancer | Original Article | France | 2020 | Ferrier et al | Absenteeism and indirect costs during the year following the diagnosis of an operable breast cancer: A prospective multicentric cohort study (6) | 6 |
| - use of chemotherapy drugs  - comorbidities  - age  - Receipt of surgery or radiation  - insurance type | Direct medical costs | 2938 breast-cancer | Original Article | USA | 2020 | Hu et al | Identifying and understanding determinants of high healthcare costs for breast cancer: a quantile regression machine learning approach (7) | 7 |
| older age, Advanced cancer stage | Direct medical costs (hospital records) | 166 patients with stage 0-III women breast cancer admitted to the hospital | Original Article | China | 2020 | Jing et al | Financial toxicity and its associated patient and cancer factors among women with breast cancer: a single‑center analysis of low‑middle income region in China (8) | 8 |
| patients who were older, not married, not obese, and not smoking tended to incur lower indirect costs. | Indirect costs (Absenteeism cost ) | 254 breast cancer | Original Article | Japan | 2020 | Ohno et al | Factors associated with humanistic burden and indirect cost among patients with cancer in Japan (9) | 9 |
| - surgery  - adjuvant therapies  - Older age  - advanced stage | Direct medical cost (Surgery, Hormonal therapy, Radiation and chemotherapy, Outpatient cost, Diagnostic test costs, Drug cost, Palliative treatment or side effect) | 1542 Patients with breast cancer | Original Article | The United States | 2020 | Mousa et al | Direct medical costs of breast cancer in Jordan: cost drivers and predictors (10) | 10 |
| - health care system factors (access to care in the public or private sector, availability of services close to home, gap payments, cost of travel)  - financial factors (impact of cancer on employment, ability to work, and career; and strategies for improving financial difficulties)  - social and community support provided by the government and not-for-profit organization | Direct medical costs | 131 patients with breast cancer | Original Article | Australia | 2020 | Slavova et al | ‘Biggest factors in having cancer were costs and no entitlement to compensation’—The determinants of out-of-pocket costs for cancer care through the lenses of rural and outer metropolitan Western Australians (11) | 11 |
| - outpatient drugs  - hospitalization  - radiology procedures | Direct medical costs | 7,032 patients with metastatic breast cancer | Original Article | The United States | 2018 | [Burudpakdee](https://ascopubs.org/author/Burudpakdee%2C+Chakkarin) et al | What are the drivers of healthcare cost among patients with metastatic breast cancer (mBC)? Total cost of care analysis to inform value-based reimbursement (12) | 12 |
| patient characteristics:  -Obesity and diabetes increase hospital charges.  -age  - smoking  Hospital characteristics:  - medium or large hospitals (large hospitals have the tendency to care for higher-risk and complex populations, with the increased need for more aggressive, costly, perioperative management.)  -income  operative characteristics:  - higher charges due to the extended operative time, and length of stay. | hospital charges | 70695 patients with breast cancer | Preclinical study | New York | 2017 | Bucknor et al | The financial impact and drivers of hospital charges in contralateral prophylactic mastectomy and reconstruction: a Nationwide Inpatient Sample hospital analysis (13) | 13 |
| - The age of the patients  - stage of tumor  - employment level of the patient  - chemotherapy | Direct medical costs | 12,580 breast cancer cases | Original Article | Italia | 2017 | Capri et al | Cost of breast cancer based on real-world data: a cancer registry study in Italy (14) | 14 |
| - stage of tumor | Direct medical costs and Direct non-medical costs | 37 hospital centers Among 2746 women with BC | Original Article | China | 2017 | [Liao](https://onlinelibrary.wiley.com/action/doSearch?ContribAuthorRaw=Liao%2C+Xian-Zhen) et al | Medical and non-medical expenditure for breast cancer diagnosis and treatment in China: a multicenter cross-sectional study (15) | 15 |
| age, income, time in survivorship from diagnosis, and use of supportive services. | Out-of-pocket (OOP) costs | 432 rural breast cancer patients | Original Article | United State | 2017 | Pisu et al | Out-of-pocket costs and burden among rural breast cancer survivors (16) | 16 |
| - Metastasis,  - facility provider affiliation  - radiation  - younger age  - hospital admissions and hospital length of stay | Direct medical costs | 5700 women  18–65 years with breast cancer | Original Article | The United States | 2017 | [Sagar](https://pubmed.ncbi.nlm.nih.gov/?sort=pubdate&size=200&term=Sagar+B&cauthor_id=28581874) et al | Cost drivers for breast, lung, and colorectal cancer care in a commercially insured population over a 6-month episode: an economic analysis from a health  plan perspective (17) | 17 |
| - extreme users of disease-monitoring testing. | Direct medical costs | 2,460 eligible patients  With breast cancer≥ 65 | Original Article | Columbia | 2016 | Accordino et al | Use and Costs of Disease Monitoring in Women with Metastatic Breast Cancer (18) | 18 |
| Chemotherapy | Direct medical costs  (inpatient, outpatient, and prescription drugs ) | 14643 women with breast cancer and over 18 years old | Original Article | The United States | 2016 | Giordano et al | Estimating Regimen-Specific Costs of Chemotherapy for Breast Cancer: Observational Cohort Study (19) | 19 |
| inpatient hospital stay  - stage of diseases  - age. | Direct medical costs  (Outpatient and inpatient treatment cost, Covered drugs cost) | 1142 breast cancer patients | Original Article | Lithuania | 2015 | Ivanauskien et al | The cost of newly diagnosed breast cancer in Lithuania (20) | 20 |
| Chemotherapy | Direct medical cost and direct non-medical cost | 201 cancer patients | Original Article | Iran | 2015 | Bahmei et al | Examination of medical and non-medical direct costs of outpatients and hospitalized cancer patients in Shiraz, Iran (21) | 21 |
| cancer clinic visits, physician billings, and hospitalizations. | Direct medical costs | 39,655 females with breast cancer | Original Article | The United States | 2014 | [Mittmann](https://www.ncbi.nlm.nih.gov/pubmed/?term=Mittmann%20N%5BAuthor%5D&cauthor=true&cauthor_uid=25489255) et al | Health system costs for stage-specific breast cancer: a population-based approach (22) | 22 |
| insurance, residence, use of outpatient services, and type of treatment. | Direct medical costs | 56 breast cancer patients | Original Article | Iran | 2014 | Kavosi et al | Catastrophic Health Expenditures and Coping Strategies in Households with Cancer Patients in Shiraz Namazi Hospital (23) | 23 |
| surgery.  - medication cost | Direct Medical Costs | 467 patients in various breast cancer stages | Original Article | Iran | 2013 | Davari et al | The Direct Medical Costs of Breast Cancer in Iran: Analyzing the Patient’s Level Data from a Cancer Specific Hospital in Isfahan (24) | 24 |
| stage at diagnosis | Direct Medical Costs (Diagnosis, Surgery, Chemotherapy, Radiation therapy, Hormone therapy,  Inpatient fee) | 129 breast cancer patient | Original Article | Vietnam | 2013 | Hoang Lan et al | Cost of treatment for breast cancer in central Vietnam (25) | 25 |
| -stage  - chemotherapy and Radiation therapy. | Direct Medical Costs | Data from databases of diagnostic, staging, and treatment modalities in breast cancer in Iran | Original Article | Iran | 2013 | [Yavari](https://www.sid.ir/en/journal/Searchpaper.Aspx?Writer=392990) et al | Health Expenditure in Initial Diagnostic and Treatment Approach to Non-Metastatic Breast Cancer in Iran (26) | 26 |
| - stage of disease  - the age of patients,  - duration of treatment | Direct Medical Costs | 301 women with breast cancer | Original Article | Australia | 1995 | [Butler](https://onlinelibrary.wiley.com/action/doSearch?ContribAuthorRaw=Butler%2C+J+R+G) et al | The costs of treating breast cancer in Australia and the implications for breast cancer screening (27) | 27 |

**Table S2. Average direct medical, direct non-medical, and indirect costs per studied breast cancer patient (USD)**

|  | **Type of costs** | **Total**  **Mean** | **% of**  **total costs** |
| --- | --- | --- | --- |
|  |  |  |  |
| **Direct medical costs** | Physicians and oncologist visits | 203.82 | 70.47 |
|  | Radiotherapy | 3085.45 |  |
|  | Chemotherapy | 1060.92 |  |
|  | Radiography | 485.25 |  |
|  | Physiotherapy | 28.33 |  |
|  | hormone therapy | 570.57 |  |
|  | Laboratory tests | 500.30 |  |
|  | Lymphedema | 41.00 |  |
|  | Hospitalization | 1915.58 |  |
|  | Medications and drugs | 416.83 |  |
|  | **Total** | **8308.02** |  |
| **Direct non-medical costs** | Accommodation | 358.66 | 19.00 |
|  | Transportation of patients and their companions | 1044.40 |  |
|  | Patients and their companions’ food | 510.14 |  |
|  | Phone and internet calls with family | 21.17 |  |
|  | Purchasing assistive devices | 17.56 |  |
|  | Babysitter and housemaid | 284.34 |  |
|  | **Total** | **2236.26** |  |
| **Indirect costs** | Patient companions’ absenteeism due to patient care | 710.18 | 10.53 |
|  | Patients’ absenteeism due to the disease | 533.41 |  |
|  | **Total** | **1243.58** |  |
| **Total Cost** |  | **11787.86** | **100** |

**References:**

1. Ahmadi F, Farrokh-Eslamlou H, Yusefzadeh H, Alinia C. Incidence of household catastrophic and impoverishing health expenditures among patients with Breast Cancer in Iran. "BMC Health Serv Res. 2021;21(1):1-9.

2. Alghamdi A, Balkhi B, Alqahtani S, Almotairi H. The economic burden associated with the management of different stages of breast cancer: a retrospective cost of illness analysis in Saudi Arabia. Healthcare. 2021;9(7):907-16.

3. Rezende LF, Ferrari G, Bahia LR, Rosa RDS, da Rosa MQM, de Souza RC, et al. Economic burden of colorectal and breast cancers attributable to lack of physical activity in Brazil. BMC Public Health. 2021;21(1):1-8.

4. Sun C-y, Shi J-f, Fu W-q, Zhang X, Liu G-x, Chen W-q, et al. Catastrophic health expenditure and its determinants among households with breast cancer patients in China: a multicentre, cross-sectional survey. Public Health Front. 2021;9(4):1-7.

5. Zhang J, Sun L. Analysis of Influencing Factors on Hospitalization Expenses of Patients with Breast Malignant Tumor Undergoing Surgery: Based on the Neural Network and Support Vector Machine. J Healthc Eng. 2021;20(1):1-7.

6. Ferrier C, Thebaut C, Lévy P, Baffert S, Asselain B, Rouzier R, et al. Absenteeism and indirect costs during the year following the diagnosis of an operable breast cancer: A prospective multicentric cohort study. J Gynecol Obstet Hum Reprod. 2021;50(6):87-101.

7. Hu L, Li L, Ji J, Sanderson M. Identifying and understanding determinants of high healthcare costs for breast cancer: a quantile regression machine learning approach. BMC Health Serv Res. 2020;20(1):1-10.

8. Jing J, Feng R, Zhang X, Li M, Gao J. Financial toxicity and its associated patient and cancer factors among women with breast cancer: a single-center analysis of low-middle income region in China. Breast Cancer Res Treat. 2020;181(2):435-43.

9. Ohno S, Chen Y, Sakamaki H, Matsumaru N, Tsukamoto K. Factors associated with humanistic burden and indirect cost among patients with cancer in Japan. J Med Econ. 2020;23(12):1570-8.

10. Mousa R, Hammad E, Melhem J, Al-Jaghbir M. Direct medical costs of breast cancer in Jordan: cost drivers and predictors. Expert Rev Pharmacoecon Outcomes Res. 2021;21(4):647-54.

11. Slavova‐Azmanova NS, Newton JC, Saunders C, Johnson CE. ‘Biggest factors in having cancer were costs and no entitlement to compensation’—The determinants of out‐of‐pocket costs for cancer care through the lenses of rural and outer metropolitan Western Australians. Aust J Rural Health. 2020;28(6):588-602.

12. Burudpakdee C, Seetasith A, Reyes CM, Ogale S, Munakata J, Chu LK. What are the drivers of healthcare cost among patients with metastatic breast cancer (mBC)? Total cost of care analysis to inform value-based reimbursement. Clin Oncol. 2018;36(15):1-6.

13. Bucknor A, Chattha A, Ultee K, Wu W, Kamali P, Bletsis P, et al. The financial impact and drivers of hospital charges in contralateral prophylactic mastectomy and reconstruction: a Nationwide Inpatient Sample hospital analysis. Breast Cancer Res Treat. 2017;165(2):301-10.

14. Capri S, Russo A. Cost of breast cancer based on real-world data: a cancer registry study in Italy. BMC Health Serv Res. 2017;17(1):1-10.

15. Liao XZ, Shi JF, Liu JS, Huang HY, Guo LW, Zhu XY, et al. Medical and non‐medical expenditure for breast cancer diagnosis and treatment in China: a multicenter cross‐sectional study. Asia Pac J Clin Oncol. 2018;14(3):167-78.

16. Pisu M, Azuero A, Benz R, McNees P, Meneses K. Out‐of‐pocket costs and burden among rural breast cancer survivors. Cancer Med. 2017;6(3):572-81.

17. Sagar B, Lin YS, Castel LD. Cost drivers for breast, lung, and colorectal cancer care in a commercially insured population over a 6-month episode: an economic analysis from a health plan perspective. J Med Econ. 2017;20(10):1018-23.

18. Accordino MK, Wright JD, Vasan S, Neugut AI, Hillyer GC, Hu JC, et al. Use and costs of disease monitoring in women with metastatic breast cancer. Clin Oncol. 2016;34(24):2820-6.

19. Giordano SH, Niu J, Chavez‐MacGregor M, Zhao H, Zorzi D, Shih YCT, et al. Estimating regimen‐specific costs of chemotherapy for breast cancer: Observational cohort study. Cancer. 2016;122(22):3447-55.

20. Ivanauskienė R, Domeikienė A, Kregždytė R, Milašauskienė Ž, Padaiga Ž. The cost of newly diagnosed breast cancer in Lithuania. Medicina. 2015;51(1):63-8.

21. Bahmei J, Rahimi H, saleh Jafari A, Habibyan M. Examination of medical and non-medical direct costs of outpatients and hospitalized cancer patients in Shiraz, Iran. Payesh (Health Monitor). 2015;14(6):629-37.

22. Mittmann N, Porter J, Rangrej J, Seung S, Liu N, Saskin R, et al. Health system costs for stage-specific breast cancer: a population-based approach. Curr Oncol. 2014;21(6):281-93.

23. Kavosi Z, Delavari H, Keshtkaran A, Setoudehzadeh F. Catastrophic health expenditures and coping strategies in households with cancer patients in Shiraz Namazi hospital. Middle East J Cancer. 2014;5(1):13-22.

24. Davari M, Mokarian F, Hosseini M, Aslani A, Nazari A, Yazdanpanah F. Direct medical costs of breast cancer in Iran; analyzing patients level data from a cancer specific hospital in Isfahan, Iran. Health Inf Manag J. 2013;10(3):9-21.

25. Hoang Lan N, Laohasiriwong W, Frederick Stewart J, Dinh Tung N, Coyte PC. Cost of treatment for breast cancer in central Vietnam. Glob Health Action. 2013;6(1):1-10.

26. Yavari P. Health expenditure in initial diagnostic and treatment approach to non-metastatic breast cancer in Iran. IRAN J BREAST DIS. 2013;6(2):14-25.

27. Butler J, Furnival C, Hart R. The costs of treating breast cancer in Australia and the implications for breast cancer screening. Aust N Z J Surg. 1995;65(7):485-91.
